# The Role of Impella in Cardiogenic Shock Complicated by an Acute Myocardial Infarction: A Meta-Analysis

**DOI:** 10.3390/jcm14020611

**Published:** 2025-01-18

**Authors:** Kiarash Sassani, Christian Waechter, Styliani Syntila, Julian Kreutz, Birgit Markus, Nikolaos Patsalis, Davide Di Vece, Bernhard Schieffer, Christian Templin, Georgios Chatzis

**Affiliations:** 1 Department of Cardiology, Angiology and Intensive Care, Philipps University Marburg, 35043 Marburg, Germany; 2 Department of Internal Medicine B (Cardiology, Angiology, Pneumology and Intensive Care Medicine), University Medicine Greifswald, 17475 Greifswald, Germany

**Keywords:** Impella, cardiogenic shock, acute myocardial infarction, infarct associated cardiogenic shock, meta-analysis, transvalvular microaxial pump

## Abstract

**Background:** Emerging evidence suggests the role of mechanical circulatory support (MCS) devices in the therapy of refractory cardiogenic shock (CS). However, largerandomized trials addressing the role of Impella in the therapy of infarct-associated CS are sparse. As such, evidence coming from comprehensive retrospective studies or meta-analyses is of major importance in order to clarify the role of the Impella device in this setting. **Methods:** Only clinical trials involving patients receiving Impella 2.5 and Impella CP for treatment of CS caused in terms of acute coronary syndrome (ACS) were included in this meta-analysis. The primary endpoint was 30-day mortality, with major bleeding and ischemic vascular complications serving as secondary endpoints. **Results:** A total of 18 observational retrospective studies (2617 patients with CS and Impella implantation) were included in this analysis. The mean age of the total participants was 64.7 ± 2.93 years. A mean mortality incidence of 45% was found between all included participants. The ischemia rate was in total 8.5 ± 4.4%, and the incidence of bleeding was 13.9 ± 5.6%. **Conclusions:** The 30-day mortality rate for patients with ACS-associated CS treated with Impella remains high. The high complication rates underline the importance of Impella use in only a very well-selected population of patients.

## 1. Introduction

Cardiogenic shock (CS) manifests as systemic hypotension resulting from cardiac dysfunction, accompanied by adequate or elevated filling pressures. This leads to insufficient tissue perfusion and eventual organ failure [1]. Mechanical circulatory support (MCS) devices, such as the Impella transvalvular microaxial pump from Abiomed Inc., are increasingly employed to alleviate afterload, enhancing cardiac reserve and ventricular metabolism to sustain hemodynamic stability. Current understanding suggests that CS disrupts circulatory dynamics, causing a severe imbalance between oxygen demand and consumption, reducing capillary density, and triggering local inflammation in microvasculature, which impairs blood flow and induces tissue edema [2]. Initial CS management entails fluid resuscitation and administration of catecholamines (inotropes and vasopressors) to maintain cardiac output (CO) and mean arterial pressure. However, escalating vasopressor doses exacerbate vasoconstriction, intensifying vascular resistance and microvascular damage, emphasizing the need for a treatment approach that considers more than just blood pressure regulation [3,4]. The Impella device presents an appealing solution. By continuously extracting blood from the left ventricle (LV), it reduces LV workload and myocardial oxygen demand [5]. Moreover, by delivering large blood volumes to the aorta, the Impella enhances mean arterial pressure (MAP) and CO while diminishing the need for catecholamines, thereby mitigating their adverse effects on microvasculature [5]. These combined effects improve systemic perfusion and coronary flow, augment CO, decrease catecholamine doses, and ultimately enhance perfusion to vital organs [5]. Despite these potential benefits, trials such as the IMPRESS in severe shock trial did not demonstrate a survival advantage with Impella over intra-aortic balloon pump (IABP) in acute myocardial infarction-related cardiogenic shock (AMI-CS) [6,7,8,9]. Our group has previously demonstrated the beneficial role of Impella in post-cardiac arrest CS [9,10,11]. Recent meta-analysis aggregating data from smaller trials yielded neutral outcomes regarding the efficacy of Impella in CS [12]. Actual guidelines recommend the use of MCS in CS with a class IIa recommendation, without, however, suggesting a special mode of escalation therapy or favoring a special device [13]. Meanwhile, the DANGER shock trial, a prospective randomized study, found no significant benefit of the device over medical treatment concerning 30-day mortality rates [14]. As such, we aimed to assess the role of Impella in AMI-related CS in terms of a systematic review and meta-analysis of the available data.

## 2. Methods

### 2.1. Studies Selection

The present study was performed according to the Cochrane Collaboration and PRISMA statements. We searched for clinical trials in The Cochrane Collaboration Central Register of Controlled Trials, EMBASE, and MEDLINE/PubMed (data as of the 25th of September 2024). We restricted our searches to human studies, clinical trials, controlled trials, or randomized trials. We used the keywords and medical subject headings “cardiogenic shock”, “Impella”, “Impella CP”, “Impella 5”, and “Impella 2.5”, as well as additional text words (such as abbreviations), in combination with an established search strategy for MEDLINE/PubMed. We also hand-searched bibliographies of identified studies, recent meta-analyses, and pulmonary hypertension guidelines. Study selection was performed by three independent reviewers (NP, KS, GC), with divergences resolved by consensus. Citations were first scanned at the title/abstract level. Shortlisted studies were then retrieved in full text. They were considered journal pre-proof suitable for inclusion if (a) reporting on a randomized control trial or observational study in which any kind of Impella device was used. In cases of studies in which different types of MCS were used, only data related to Impella were considered; (b) CS was the indication. Studies were excluded if (a) they included less than 50 patients treated with Impella, (b) the indication was high-risk PCI without CS, or (c) pediatric populations or congenital heart disease were involved. Corresponding authors of each study were asked to provide more data of their respective paper if needed. Key study and patient characteristics were extracted, including age, gender, cardiovascular risk factors, clinical presentation, ejection fraction, lactate levels, and use of inotropic medication. Impella 5.5 cases were excluded from our analysis, as this study aimed to evaluate the effect of Impella in emergency and refractory cardiogenic shock scenarios requiring immediate intervention. The objective was to reflect real-world data and clinical practice, particularly in settings where the mandatory presence of a vascular or cardiothoracic surgeon—often unavailable in emergencies or non-tertiary centers—was not feasible.

### 2.2. Endpoints and Definitions

The primary endpoint was defined as 30-day mortality. The secondary endpoint was vascular access complication and major bleeding. Thirty-day mortality was considered as thirty-day all-cause death. Major vascular access complications were defined as critical limb ischemia requiring Impella removal or vascular/surgical intervention. Major bleeding was considered as bleeding BARC > 2, TIMI major, or GUSTO severe bleeding. The quality of included studies was independently appraised by 2 reviewers (KS and GC), with disagreements resolved by consensus. For each included paper, we evaluated the risk of bias (low, unclear, or high) for random-sequence generation, allocation concealment, blinding of patients and physicians, blinding during assessment of follow-up, incomplete outcome evaluation, and selective reporting, in keeping with the Cochrane Collaboration approach.

### 2.3. Statistical Analysis

Continuous variables are reported as mean (SD) or median (first and third quartile). Categorical variables are expressed as n (%). Statistical pooling for incidence estimates was performed according to a random-effect model with generic inverse-variance weighting, computing risk estimates with 95% confidence intervals (CIs) using R Project for statistical computing (Version 4.4.1). Small study bias was appraised by graphical inspection of funnel plots. Meta-regression analysis and leave-one-out analysis were performed to assess the impact of baseline features on the primary endpoint with Comprehensive Meta-analysis software (trial version). Hypothesis testing for statistical homogeneity was set at the 2-tailed 0.10 level and based on the Cochran Q test, with I^2^ values of 25%, 50%, and 75% representing mild, moderate, and severe heterogeneity, respectively.

## 3. Results

**Figure 1:**
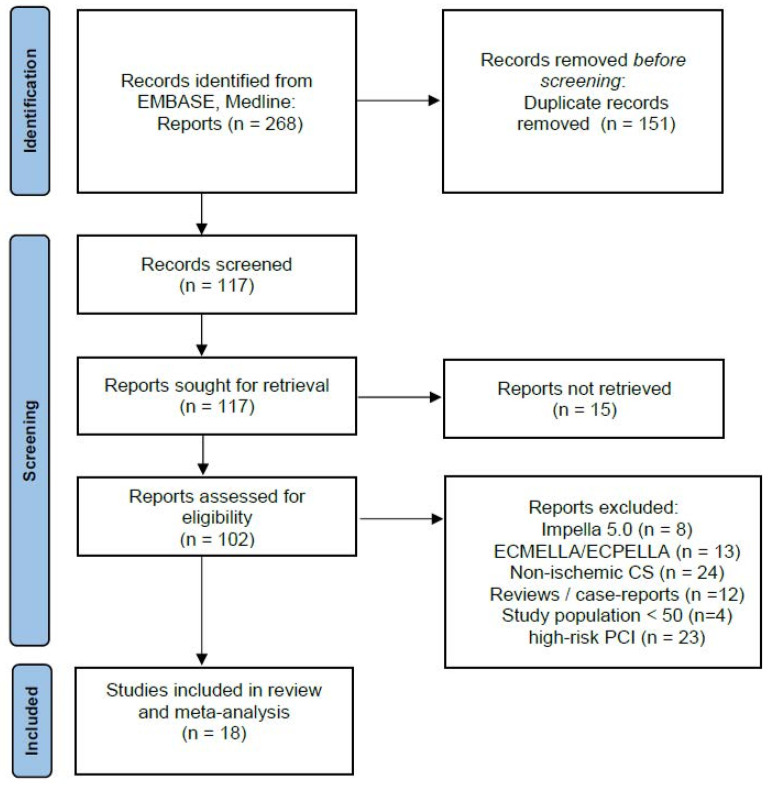
Flow chart of the included studies.

**Figure 2:**
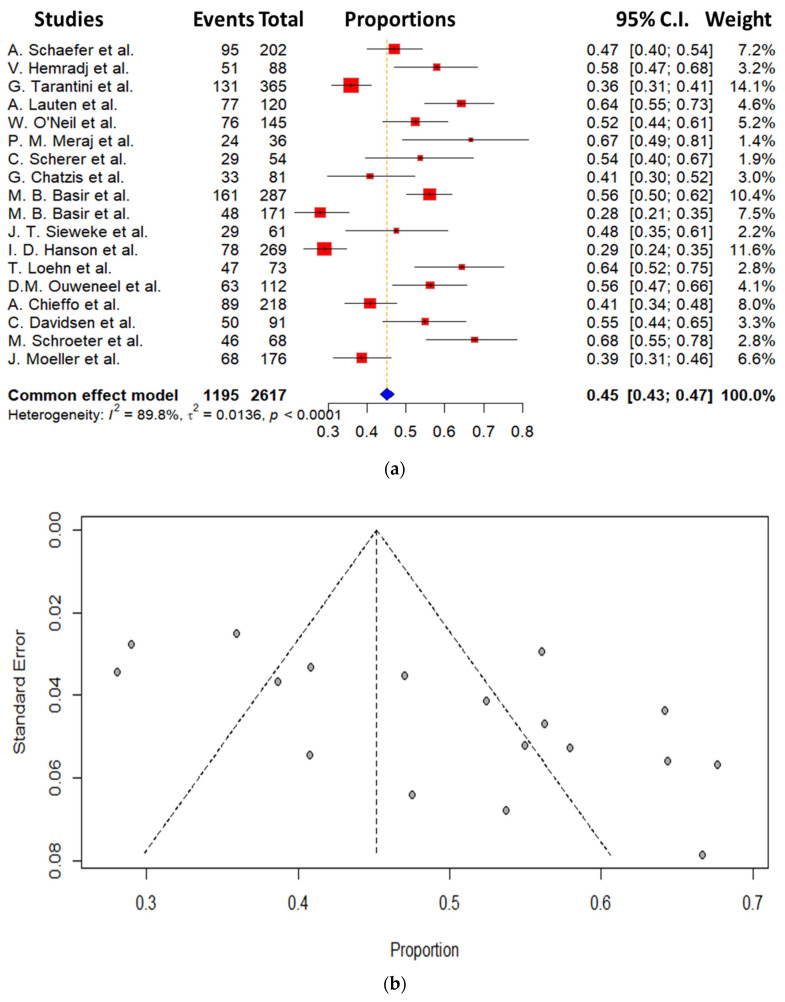
(**a**) Effect of Impella on 30-day mortality. (**b**) Funnel plot of Impella on mortality [10,14,15,16,17,18,19,20,21,22,23,24,25,26,27,28,29,30].

A total of 18 observational retrospective studies (2617 patients with CS and Impella implantation) were included in this analysis (see Figure 1 and Table 1). The mean age of the total participants in all studies was 64.7 ± 2.93 years; data about prior resuscitation were not present in all of the studies; however, the percentage varied from 23% to 74%, implying a relatively ill cohort of patients. Lactate levels upon admission were also unfortunately not available in all studies; however, in most of them, the lactate levels varied from 4.7 to 8.6 mmol/lit, depicting a profound state of shock as well. As far as the ejection fraction as a direct measurement of the LV function upon admission is concerned, the available data from the included studies revealed a severe impairment of the LV functionality with values varying from 25 to 34%. Sufficient data about Impella implantation according to the coronary intervention made were unfortunately not available, so we avoided an additive comparison between pre- and post-PCI groups. Main baseline characteristics of the studies included are listed in Table 2. We could show as a major finding of our study a mean mortality incidence of 45% from all participants, with rates ranging from 28% to 67%. This mortality rate refers to 30 days from initial admission, which may explain the relatively high mortality incidence despite the use of mechanical support with an Impella. The mortality rates related to the specific studies included in this meta-analysis are presented in Figure 2a,b.

### Secondary End Points

**Figure 3:**
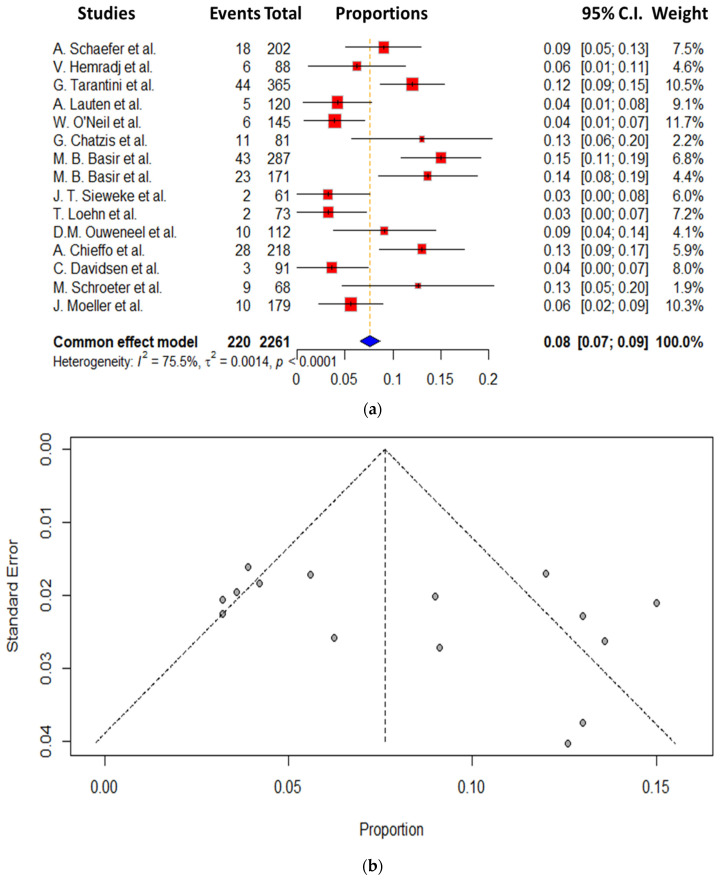
(**a**) Forest plot of Impella on ischemia complication. (**b**) Funnel plot of Impella on ischemia complication [10,14,15,16,17,18,19,20,22,24,25,26,28,29,30].

As secondary endpoints in this study, we concentrated on the bleeding and ischemia incidence of the Impella use in these studies. Unfortunately, available data from all studies were not present; however, we could extract these results in the vast majority of the studies included. The ischemia and bleeding incidence was available in 15 of the 18 studies included in the present meta-analysis, corresponding to a total of 2261 patients from the total of 2617 participants (86.4% of the total cohort). The ischemia rate was in total 8.5 ± 4.4%, ranging from 3.6 to 15%. The lowest incidence of ischemia was referred to in the publication of Lauten et al. with 3.4%, where only Impella 2.5 was used, whereas the highest was referred to in the study of Basir et al., with an incidence of 15%, where both Impella systems were used. The incidence of the ischemia in this meta-analysis, as well as the funnel plot of this complication, are presented in Figure 3a,b [15,20].

**Figure 4:**
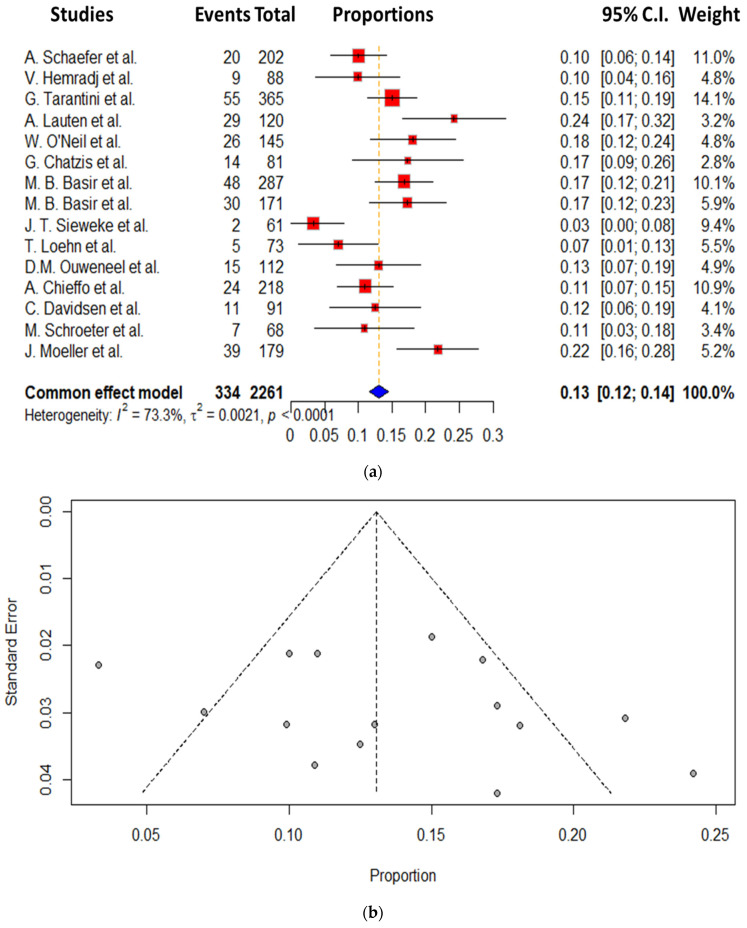
(**a**) Effect of Impella on bleeding complication. (**b**) Funnel plot of Impella on bleeding complication [10,14,15,16,17,18,19,20,22,23,24,25,26,27,28,29,30].

As far as the bleeding complication is concerned, we demonstrated a total incidence of 13.9 ± 5.6%. The bleeding rates ranged from 3.3 to 24.2%, with the lowest being reported by Lauten et al. and the highest from Sieweke et al. The detailed data concerning bleeding complications are presented in Figure 4a,b [20,29].

## 4. Discussion

The primary findings of our meta-analysis are as follows:

1. The 30-day mortality rate for CS patients is still high even with the use of Impella.
2. Our findings do, however, indicate that vascular complications remain high, being, however, quite heterogeneous between the studies.

Since the introduction of percutaneous coronary intervention, there have not been any notable improvements in CS outcomes pertaining to any specific materials like dedicated medication, applied procedures, or specific conventions [31]. Although this technology improved the short-term outcome of the treated patients, the mortality remained very high, so further devices were developed in order to improve the outcome.

Several randomized trials did not show a prognostic benefit of pressure-unloading devices for the left ventricle like IABP [32], whereas devices to reduce the volume of the left ventricle with Impella appeared to be promising due to improving the prognosis [33]. According to our data, patients with CS treated by Impella had a 30-day mortality rate that is consistent with previous studies [12,31,34]. Based on the decreased risk of multiple organ failure and the transition to cardiometabolic shock, these data suggested that an increase in cardiac output by applying these procedures may improve prognosis and that early device initiation may improve survival. When compared to other available data, the mortality rate in our study was not lower. There may be several reasons for this observation. We examined datasets containing different ratios of Impella CP and 2.5. A low increase in CO is likely the reason why Impella 2.5 is not associated with better outcomes, according to other studies [35]. As previously indicated, the patient cohorts included in this meta-analysis were limited to those who experienced ACS-related CS, which is associated with a higher rate of hospital discharge than acute myocardial infarction without CS. Certain complex factors, such as the occurrence of CS prior to or following Impella implantation, the beginning point of the coronary intervention with the corresponding outcome upon TIMI flow achievement, and the fact of the presence of cardiopulmonary resuscitation in a part of these patients in CS with a delay of return of spontaneous circulation (ROSC), must be taken into account when analyzing these outcomes. In a similar previous meta-analysis of Impella in CS, Iannaccone et al. demonstrated a 30-day mortality of 48% [35]. However, in this study, ACS was not the unique cause of CS. Moreover, patients were also treated with Impella 5.0, which can, of course, offer more support in the case of CS, even though mortality was higher than in our population. The operator’s expertise plays a pivotal role in the success of Impella-assisted interventions, influencing both the quality of the procedure and the speed at which results are achieved. This underscores the importance of technical proficiency and experience in optimizing outcomes for patients undergoing mechanical circulatory support interventions. Furthermore, the management of metabolic acidosis, pulmonary failure, and biventricular insufficiency, which may require increased support and partially direct arterial oxygenation—possibly provided by extracorporeal membrane oxygenation—all play significant roles in the outcome for this group of patients during the subsequent intensive care management of the overall course. Because mortality may be significantly impacted by each of these variables, a complex interaction between these factors rather than Impella’s lack of effect could account for our findings. In this context, some biases need to be discussed. First of all, the effect of confounding must be mentioned since the combination of the factors mentioned above may influence the target mortality rate differently than each individual factor on its own in the sense of synergism with an additive or even multiplicative effect. Secondly, effect modification by factors such as gender and age must be considered, which cannot be described by either a multiplicative or an additive model but follow their own biological laws. Thirdly, the effect of publication bias must be taken into account, as there may be studies that would be suitable for inclusion in this meta-analysis from a qualitative point of view but which have not been published so far or have only been published in unknown journals. The symmetrical funnel plots concerning all endpoints, that is, mortality, bleeding, and ischemia, imply a low possibility of systematic bias. In addition, this meta-analysis also included multicenter studies. Unfortunately, we do not know to what extent the inclusion and exclusion criteria and the treatment and diagnostics differed in these studies. It is important to emphasize that meta-regression analysis, rather than direct comparisons, is the foundation for our findings. The high heterogeneity of our results depicts real-world clinical practice, since data stemming from various observational studies produced on the basis of various clinical algorithms and missing guideline-directed therapies can rarely present any kind of homogeneity. Even after sensitivity or leave-out analyses, the heterogeneity remained high (Supplementary Material Figures S1–S3).

The device-related complications are another significant area in which our meta-analysis sheds additional light. The rates of major vascular ischemic complications and major bleeding were 8% and 13%, respectively. These outcomes align with earlier research data [35]. Possible explanations for these results include the following conditions: The opportunity for a dedicated access assessment is compromised when comparing the elective procedure to the urgency setting, which is typically present in a cardiogenic shock scenario. This is because choosing the appropriate sheet to femoral artery ratio is crucial given the use of large French sizes with Impella. Here too, the experience of the operator with regard to puncture and correct handling of larger access routes plays a decisive role. The inclusion of these vascular complications in the analysis could only explain the high rate of motility in the setting of ACS with CS. The higher rate of complications in the multicenter studies with larger cohorts was likely caused by the inclusion of centers with less experience using Impella and by the larger number of sites, which may have affected the results [25].

### 4.1. Study Limitations

This is not a patient-generated data analysis; rather, the data utilized came from observational retrospective studies, so the results are to be considered just as hypothesis and not as solid evidence of causality. Given the literature review nature of this study, we opted not to register the analysis in specialized databases, such as PROSPERO. An important limitation of our study is the high heterogeneity between the studies included. However, our analysis aimed to provide insight into real-world data and extract feasible outcomes without any comparisons, mainly based on observational studies. The precise time latency between the onset of CS and Impella implantation, as well as the precise timing of the coronary intervention, could not be found in all the studies included in this analysis, so the significant differences between the studies may result from different interclinical procedure algorithms. Moreover, the missing data on the type of Impella (2.5 or CP) used as well as on the type of ACS, such as STEMI or NSTEMI, in the majority of studies used in the present meta-analysis could not make possible a comparison between these subgroups in order to identify possible favorable effects from additional support or eventually more complications due to larger bore access needed without net clinical benefit. To further assess the influence of the lactate level, the extension of coronary disease (e.g., multi-vessel disease, left main disease, or graft disease), ejection fraction, or the inotropes that may have a main impact on the results, meta-regressions involving sufficient data were not available from all of the included studies. Even still, our study is, to the best of our knowledge, the first study to assess the role of Impella in the unique case of ACS-related CS in terms of systematic review and meta-analysis.

### 4.2. Future Perspective

Possible aspects for improving the outcome in patients with ACS complicated with CS could be the selection of the right device in the right patient with timely implantation and implementation of ubiquitous procedural steps in the form of shock team networks in dedicated centers to firstly ensure maximum safety for the patient and secondly to achieve better comparability of the acquired data for future studies to better understand the research of this clinical setting.

## 5. Conclusions

The findings of this meta-analysis indicate that the use of the Impella device in the management of cardiogenic shock demonstrates a mortality rate of approximately 50%, comparable to that observed with medical therapy alone. These results underscore the need for cautious interpretation of its benefits in this critically ill population. The lack of a clear survival advantage highlights the importance of individualized patient selection and underscores the need for further high-quality randomized controlled trials to better define its role. Adjunctive strategies focusing on timely intervention, improved patient selection, and comprehensive care pathways may be critical to optimizing outcomes in patients with cardiogenic shock.

## Figures and Tables

**Table 1:** Included studies.

Author	Publication Date	Journal	Country	Study Design	Clinical Endpoint
B. Basir [15]	2017	AJC	USA	Retrospective	30 days mortality and overall survival to discharge
B. Basir [16]	2019	CCI	USA	Prospective Multicenter	In hospital survival and 30 days mortality
Chatzis [10]	2021	CCM	USA	Retrospective	Overall survival to discharge and at 6 months
Chieffo [17]	2020	EI	Italy	Retrospective Multicenter	All-cause mortality at hospital and at one year
Davidsen [18]	2019	ERC	Norway	Retrospective	30 days outcome
Loehn [19]	2018	ACC-EHJ	Germany	Retrospective	Survival at discharge, 30 and 90 days
Lauten [20]	2012	CHF	Europe	Retrospective Multicenter	30 days mortality
Hanson [21]	2020	CCI	USA	Prospective blinded	Survival to discharge and 30 days mortality
Hemradj [22]	2020	PLO	Netherlands	Retrospective	30 days mortality
M. Meraj [23]	2017	JIC	USA	Retrospective	30 days mortality
O`Neil [24]	2014	JIC	USA	Retrospective	Survival to discharge and 30 days mortality
Ouweneel [25]	2018	EHJ	Netherlands	Retrospective	30 days mortality and 6 months mortality
Schäfer [26]	2021	FCM	Germany	Retrospective	30 days mortality
Scherer [27]	2020	JCM	Germany	Retrospective	1 year mortality
Schroeter [28]	2016	JIC	Germany	Retrospective	1 year mortality
Sieweke [29]	2018	EHJ	Germany	Prospective	30 days mortality
Tarantini [30]	2020	CCI	Italy	Retrospective	1 year mortality
Moeller [14]	2024	NEJM	Denmark Germany	Prospective Multicenter	Survival on 6 months

AJC: *American Journal of Cardiology*, CCI: Catheter Cardiovasc Intervention, CCM: Critical Care Medicine, EI: Euro Intervention, ERC: European Resuscitation Council, ACC-EHJ: Acute Cardiovascular Care—*European Heart Journal*, AJC: *American Journal of Cardiology*, CHF: Circulation Heart Failure, CCI: Catheter Cardiovasc Intervention, PLO: PLoS One, JIC: *Journal of Interventional Cardiology*, CHF: Circulation Heart Failure, PLO: PLoS One, JIC: *Journal of Interventional Cardiology*, EHJ: *European Heart Journal*, FCM: Front Cardiovasc Medicine, JCM: *Journal of Clinical Medicine*, NEJM: *New England Journal of Medicine*, USA: United States of America.

**Table 2:** Included studies’ baseline characteristics.

Author	No. of Patients	30-Day Mortality (%)	Ischemic Events (%)	Lactate (mmol/L)	Age (Years)	Bleeding (%)	LVEF (%)	Prior CPR (%)	pH
B. Basir [15]	287	56%	5%	5.8	66	17.0%	22%	52%	n.a.
B. Basir [16]	171	28%	13.6%	5.4	63	17.3%	n.a.	49%	n.a.
Chatzis [10]	81	41%	13%	8.6	68	17%	33%	n.a.	n.a.
Chieffo [17]	218	41%	13%	6.1	64	11%	35%	n.a.	7.3
Davidson [18]	91	55%	3.6%	n.a.	61	12.5%	26%	74%	n.a.
Loehn [19]	73	64%	3.2%	6.3	69	7%	29%	43%	7.3
Lauten [20]	120	64%	4.2%	5.8	64	24%	27%	41%	n.a.
Hanson [21]	269	29%	n.a.	n.a.	64	n.a.	n.a.	n.a.	n.a.
Hemradj [22]	88	58%	6.25%	5.1	60	9.9%	n.a	58%	7.2
M. Meraj [23]	36	36%	3%	4.7	70	3%	25%	44%	n.a.
O`Neil [24]	145	52%	3.9%	4.1	64	18%	26%	23%	n.a.
Ouweneel [25]	112	56%	9.1%	6.2	60	13%	n.a.	60%	7.2
Schäfer [26]	202	47%	9%	5.2	66	9%	26%	n.a.	n.a.
Scherer [27]	54	54%	5%	3.4	67	23%	n.a.	51%	n.a.
Schroeter [28]	68	55%	12.6%	n.a.	63	10.9%	27%	n.a.	n.a.
Sieweke [29]	61	48%	3.2%	n.a.	62	3.3%	n.a.	n.a.	n.a.
Tarantini [30]	365	36%	12%	4.7	66	15%	25%	n.a.	7.4
Moeller [14]	179	39%	10%	4.6	67	22%	25%	22%	n.a.

LVEF: left ventricular ejection fraction; CPR: cardiopulmonary resuscitation; n.a.: not available.

## Data Availability

The data that support the findings of this study are available from the corresponding author upon reasonable request.

## Supplementary Materials

The following supporting information can be downloaded at: https://www.mdpi.com/article/10.3390/jcm14020611/s1. Figure S1: Funnel plot of mortality after sensitivity analysis. Figure S2: Funnel plot of ischemia complication after sensitivity analysis. Figure S3: Funnel plot of bleeding compplication after sensitivity analysis.
