# Supplementary figures and images for: The Role of Impella in Cardiogenic Shock Complicated by an Acute Myocardial Infarction: A Meta-Analysis
Source: J Clin Med. 2025 Jan 18;14(2):611. doi: 10.3390/jcm14020611 (PMC11766096; doi:10.3390/jcm14020611)

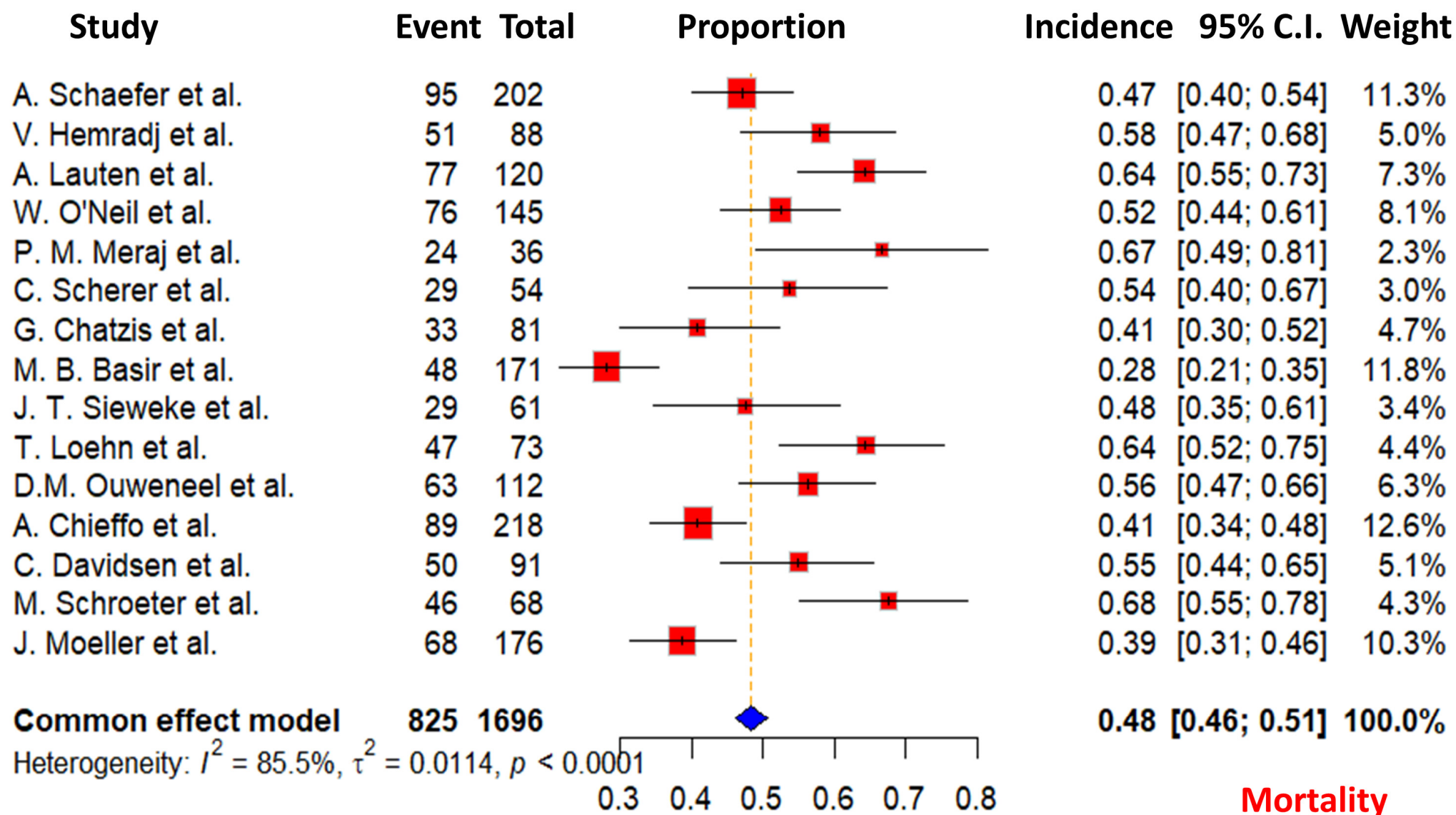

Supplement: Supplementary file 1 [file jcm-14-00611-s001.zip › Figure_S1._Mortality.pdf]

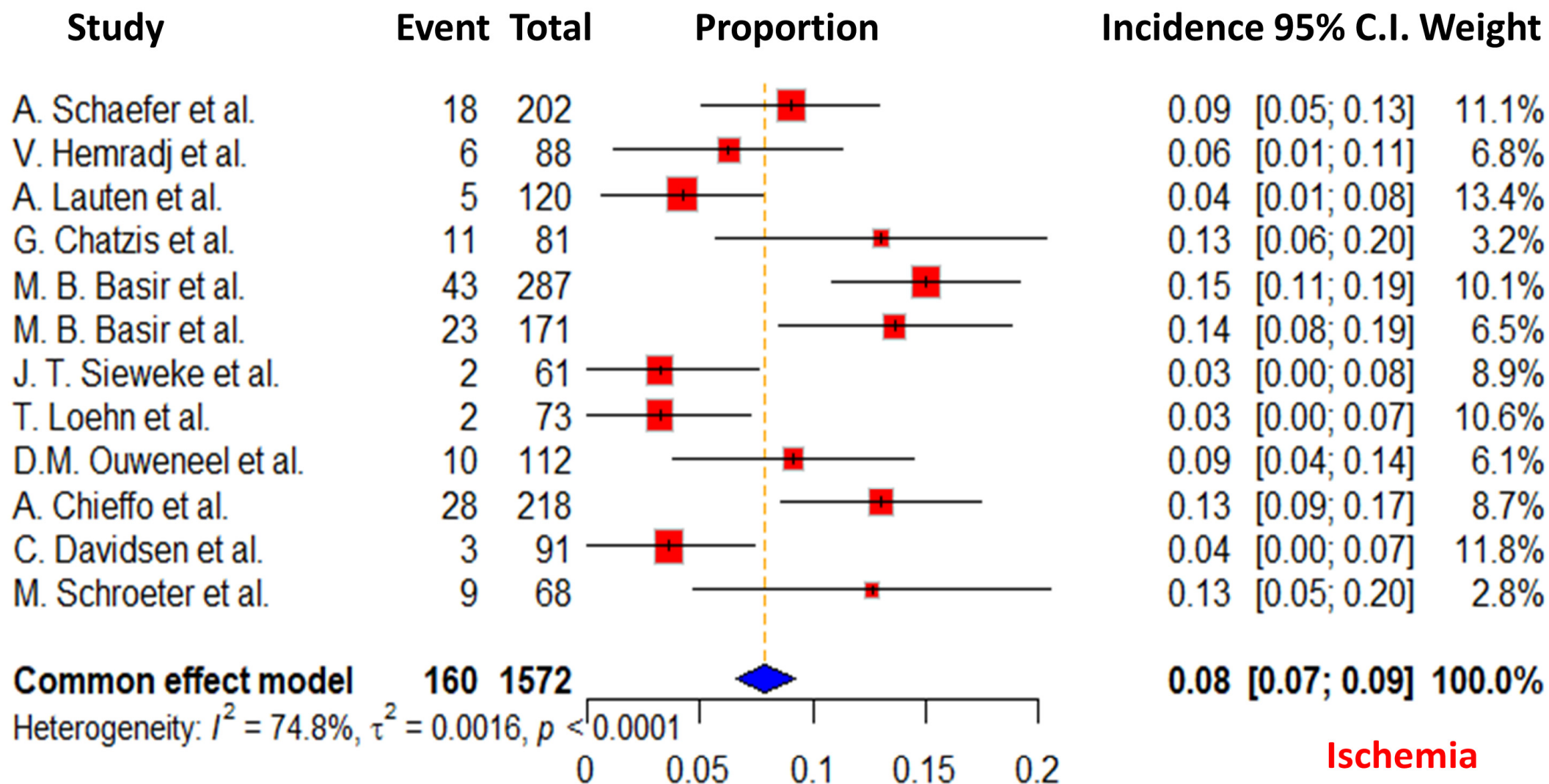

Supplement: Supplementary file 1 [file jcm-14-00611-s001.zip › Figure_S2._Ischemia.pdf]

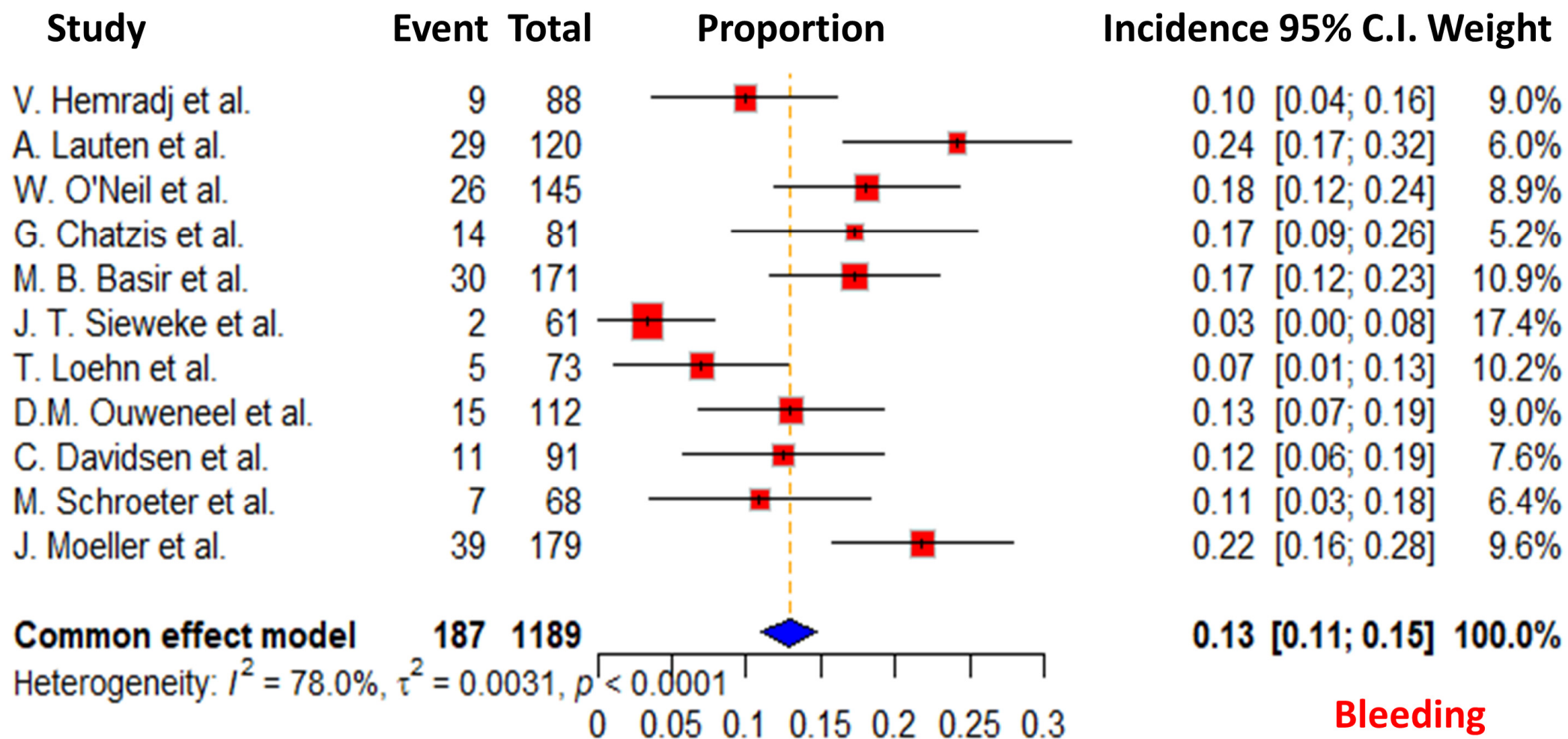

Supplement: Supplementary file 1 [file jcm-14-00611-s001.zip › Figure_S3._Bleeding.pdf]
